# Reduced Bik expression drives low-grade airway inflammation and increased risk for COPD in females

**DOI:** 10.1172/JCI177753

**Published:** 2024-02-15

**Authors:** Irina Petrache, David W.H. Riches

**Affiliations:** 1 Division of Pulmonary, Critical Care and Sleep Medicine, Department of Medicine, National Jewish Health, Denver, Colorado, USA.; 2 Division of Pulmonary Sciences and Critical Care Medicine, Department of Medicine, University of Colorado School of Medicine, Aurora, Colorado, USA.; 3 Program in Cell Biology, Department of Pediatrics, National Jewish Health, Denver, Colorado, USA.; 4 Department of Research, Veterans Affairs Eastern Colorado Health Care System, Denver, Colorado, USA.

## Abstract

Chronic low-grade inflammation is increasingly recognized as a subtle yet potent risk factor for a multitude of age-related disorders, including respiratory diseases, cardiovascular conditions, metabolic syndromes, autoimmunity, and cancer. In this issue of the *JCI*, Mebratu, Jones, and colleagues shed new light on the mechanisms that promote low-grade airway inflammation and how this contributes to the development of chronic obstructive pulmonary disease (COPD). Their finding that Bik deficiency leads to spontaneous emphysema in female mice, but not in males, marks a notable advancement in our understanding of how inflammatory processes can diverge based on biological sex. This finding is of clinical relevance, given the vulnerability of women to developing COPD.

## Low-grade inflammation and COPD development in women

While a rapid, robust inflammatory response represents an integral component of innate mammalian host defense mechanisms against pathogens, active resolution of inflammation is required for the homeostatic return to normal tissue structure and function. Persistent low-grade inflammation due to impaired resolution mechanisms, unremitting activation of proinflammatory pathways, or both have the potential to contribute to the pathogenesis of multiple diseases, including the highly prevalent cardiovascular disease, metabolic syndrome, and chronic obstructive pulmonary disease (COPD) (1–4). Since 2008, the prevalence of COPD among women has equaled that of men, partly due to increased tobacco use among women worldwide and exposure to biomass fuels (5). This demographic shift is accompanied by unique clinical presentations in women, including more pronounced dyspnea, a tendency toward anxiety and depression, undernutrition, a higher incidence of non–small cell lung cancer (particularly adenocarcinoma), and osteoporosis. Moreover, COPD substantially affects women’s quality of life more than that of men, and women with COPD face greater underdiagnosis and fewer spirometry tests and medical consultations, highlighting a need for more gender-specific research and approaches in treating COPD (6). In this issue of the *JCI*, Mebratu, Jones, et al. (7) identify a pathway leading to low-grade inflammation in the airways that leads to the spontaneous development of emphysema in female mice. This study sheds important light on a mechanism controlling low-grade inflammation and on the increasing susceptibility and prevalence of COPD in women (as recently reviewed in ref. 6).

**Figure 1:**
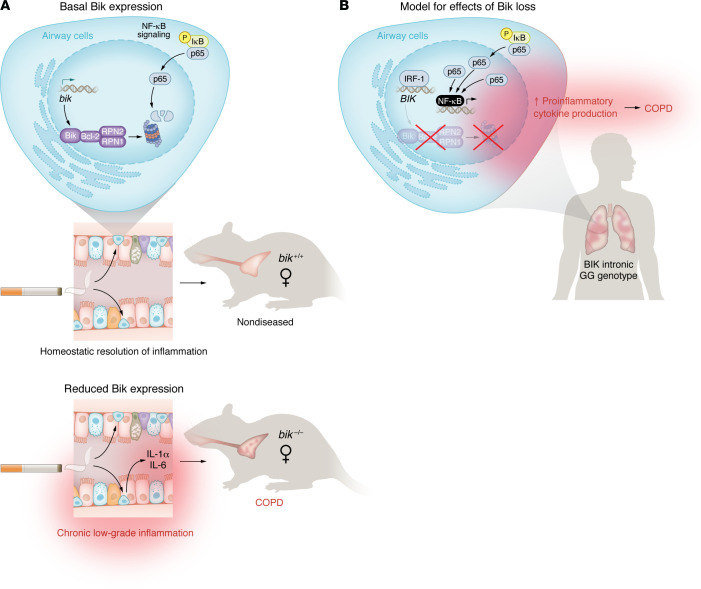
Reduced Bik expression in airway epithelial cells preferentially promotes COPD development in females. (**A**) A reduction in Bik expression in airway epithelial cells promotes chronic low-grade lung inflammation, leading to emphysema in female mice. Cigarette smoke has been shown to reduce Bik expression in airway epithelial cells. (**B**) Mechanistic pathway leading to increased proinflammatory gene expression in the context of reduced Bik expression involves the impaired recruitment of nuclear proteasomes, leading to increased nuclear p65 levels and NF-κB activation.

Mebratu, Jones, et al. highlight the importance of Bik, a proapoptotic Bcl-2 family member protein, in modulating low-level inflammation. The authors have identified a role for Bik in suppressing proinflammatory NF-κB activation. They show that, when the expression of Bik in airway epithelial cells was reduced, as occurs in COPD patients and in human airway epithelial cells following exposure to cigarette smoke (8), airway inflammation was augmented and substantial alveolar loss, a hallmark of emphysema development, ensued. Using a transgenic complementation approach in which Bik could be inducibly expressed in airway epithelial cells in *bik^–/–^* mice, the authors found that lung inflammation and emphysema were markedly reduced. The results suggest that low levels of Bik expression in airway epithelial cells, and the consequential increase in low-grade airway inflammation, are causally linked to alveolar loss during emphysema development in female mice (Figure 1A).

This finding is particularly intriguing, as it not only elucidates a function of Bik, but also underscores a potentially complex sex-specific interplay between apoptosis-regulating proteins and inflammation. To begin to address the underlying mechanisms, the authors tested the hypothesis that Bik expression dampens inflammation through the suppression of NF-κB, the archetypal proinflammatory signaling pathway. They initially found that cytosolic fractions of female airway epithelial cells from *bik^+/+^* or *bik^–/–^* mice contained similar quantities of IκBα in a complex with the NF-κB p65 subunit and similar levels of IκBα degradation. However, nuclear NF-κB p65 levels were elevated only in epithelial cells isolated from female *bik^–/–^* mice, suggesting that reduced Bik expression somehow led to the enhanced accumulation of p65 in nuclei, independently of IκBα degradation. The authors found interferon regulatory transcription factor-1 (IRF-1) was a key transcriptional suppressor of Bik expression, thereby reducing nuclear p65 levels. Taken together, these studies suggest that Bik controls the activation of NF-κB through a mechanism that regulates nuclear p65 levels (Figure 1B).

The primary interaction partner of Bik is Bcl-2, an antiapoptotic protein that has also been shown to dampen NF-κB activation (9). Although Bik is predominantly localized to, and interacts with, Bcl-2 in the endoplasmic reticulum via its BH3 domain, it was plausible that a heteromeric Bik/Bcl-2 protein complex might somehow be involved in regulating nuclear p65 levels. Using a combination of proteasomal inhibitors as well as the Bcl-2 inhibitor ABT-263, which blocks interactions between Bcl-2 and BH3 domain proteins, along with a systematic proteomic analysis of the Bik and Bcl-2 interactome, the authors showed that Bik modification of Bcl-2 led to heterocomplex formation and localization to the inner aspect of the nuclear membrane, wherein the Bik/Bcl-2 complexes recruited Rpn1 and Rpn2 and assembled nuclear proteasomes (Figure 1B).Thus, in the presence of Bik, tonic proteasomal degradation of nuclear p65 keeps NF-κB activation in check, whereas in the setting of Bik deficiency, as seen in airway epithelial cells in COPD, NF-κB activation is increased (Figure 1B).

The sex-specific aspect of these findings is especially noteworthy. The study’s observation that females, both mice and humans, are more susceptible to Bik-related inflammatory responses and lung tissue damage provides a crucial insight into sex-specific differences in disease susceptibility and progression. To begin to unveil the mechanisms of Bik-dependent sex-autonomous effects, the authors showed that primary airway epithelial cells from female wild-type mice exhibited lower levels of Bik and Bcl-2 compared with those from male mice and that, rather than being under the control of female sex hormones, Bik and Bcl-2 expression might be controlled genetically. While additional studies are clearly needed, this finding could be pivotal in shaping future research directions and therapeutic strategies.

## Clinical implications

The authors’ discovery of a single nucleotide polymorphism in the human Bik (*BIK*) gene (an intronic A-G transversion in the putative promoter region in which the GG genotype functionally mirrors Bik deficiency seen in mice) adds a layer of translational relevance to the findings (Figure 1B). The authors found that subjects with the GG genotype displayed increased inflammation and accelerated lung function decline. This finding in humans underscores the possibility of personalized medical approaches tailored to individuals’ genetic profiles. Although the authors were unable to detect differences between genotype when the data were stratified by sex, they found that older subjects with the GG genotype displayed a greater decline in FEV1 compared with matched subjects bearing the AA genotype. Furthermore, subjects with the GG genotype preferentially bound the transcriptional regulator IRF-1, which suppressed Bik expression and was associated with higher levels of circulating proinflammatory cytokines compared with those of individuals with the AA genotype. Taken together, these genetic and mechanistic studies in humans suggest that reduced Bik expression results in low-grade inflammation and reduced lung function in patients with COPD.

## Limitations and future directions

The findings reported by Mebratu, Jones, et al. (7) provide insights into why women are more susceptible to COPD than men and provide mechanistic answers to the question of how alterations in airway cell function can contribute to the loss of alveoli in emphysema. These investigations built on previous comparative studies identifying sex-specific endotypes in COPD. For example, COPD females have unique disease-specific CD4^+^ T helper and CD8^+^ T cytotoxic cell responses (10), alterations in leptin metabolism with increased leptin secretion (11), association between adipose tissue and inflammatory markers (12), and increased fatty acid–binding protein 4, a lipid chaperone that regulates inflammation (13). Inevitably, however, the Mebratu, Jones, et al. (7) study spawns additional questions. Given the striking lung phenotype seen in female mice, it remains to be determined whether the functional polymorphism in the first intronic region of the human *BlK* gene will also be found to be associated with decreased lung function in women, especially smokers. Future studies with larger cohorts should be able to provide an answer to this question. Additionally, while the elegant experiments in which Bik expression in airway epithelial cells was found to rescue the inflammatory and emphysematous phenotype in *bik^–/–^* mice, this conclusion might be strengthened in mice bearing floxed *Bik* alleles, since loss of Bik in other cells, e.g., inflammatory cells, might also indirectly contribute to the phenotype of whole-body *bik^–/–^* mice. Finally, the major roles of Bik as an inducer of mitochondrial apoptosis (14–16) and of Bcl-2 in the regulation of apoptosis and autophagy (17), processes found pathogenic in emphysema and COPD, remain to be explored in the context of the interaction of the Bik/Bcl-2 axis reported here.

Finally, the therapeutic potential of transgenic Bik expression in mitigating allergen or LPS-induced lung inflammation and reversing emphysema in female mice could pave the way for novel treatments targeting low-grade inflammation, a common but often overlooked contributor to chronic diseases. Indeed, the work reported by Mebratu, Jones, et al. in this issue of the *JCI* (7) not only advances our knowledge of the pathogenesis of COPD, but has potentially important implications for multiple other human diseases linked to ongoing and age-related low-level inflammation.

## Version 1 02/15/2024

Electronic publication
